# Host phylogeny, diet, and habitat differentiate the gut microbiomes of Darwin’s finches on Santa Cruz Island

**DOI:** 10.1038/s41598-019-54869-6

**Published:** 2019-12-11

**Authors:** Wesley T. Loo, Jefferson García-Loor, Rachael Y. Dudaniec, Sonia Kleindorfer, Colleen M. Cavanaugh

**Affiliations:** 1 Department of Organismic and Evolutionary Biology, Harvard University, Cambridge, MA USA; 2 Laboratory of Evolutionary Biology, San Francisco University, Quito, Ecuador; 3 Department of Biological Sciences, Macquarie University, Sydney, NSW Australia; 4 College of Science and Engineering, Flinders University, Adelaide, SA Australia; 5 Konrad Lorenz Research Center for Behaviour and Cognition and Department of Behavioural Biology, University of Vienna, Vienna, Austria

**Keywords:** Coevolution, Microbiome, Symbiosis

## Abstract

Darwin’s finches are an iconic example of an adaptive radiation with well-characterized evolutionary history, dietary preferences, and biogeography, offering an unparalleled opportunity to disentangle effects of evolutionary history on host microbiome from other factors like diet and habitat. Here, we characterize the gut microbiome in Darwin’s finches, comparing nine species that occupy diverse ecological niches on Santa Cruz island. The finch phylogeny showed moderate congruence with the microbiome, which was comprised mostly of the bacterial phyla Firmicutes, Actinobacteria, and Proteobacteria. Diet, as measured with stable isotope values and foraging observations, also correlated with microbiome differentiation. Additionally, each gut microbial community could easily be classified by the habitat of origin independent of host species. Altogether, these findings are consistent with a model of microbiome assembly in which environmental filtering via diet and habitat are primary determinants of the bacterial taxa present with lesser influence from the evolutionary history between finch species.

## Introduction

All living organisms evolve in a microbial world and form relationships with microbes resulting in a characteristic microbiome^1^. The composition of the vertebrate gut microbiome has direct impacts on host health and function, from effects on nutrition^2^ and metabolism^3^, to host behavior^4^ and immune function^5^. While diet is known to influence microbiome composition in a variety of vertebrate and invertebrate taxa^6–8^, host phylogeny can also be a key determinant^9^. Broad-scale surveys of avian microbiomes have found that taxonomic and phylogenetic relationships correlate more strongly with microbiome composition compared to life history traits and ecological variables in neotropical and old world passerines^10,11^. Adaptive radiations provide the opportunity to investigate evolutionary and ecological diversification processes within closely related species, and offer increased resolution to disentangle the mechanisms driving microbiome-host phylogenetic relationships. Typically, species in adaptive radiations evolve to occupy different ecological niches and may subsequently occur in sympatry where they exhibit varying diets and behaviors^12^. Our understanding of how these important microbial communities are affected by host biology and evolution is best studied in a species system with a well-defined phylogeny and ecology.

Darwin’s finches in the Galápagos archipelago offer a natural experiment for investigating the effects of host phylogeny, diet, and habitat on the bacterial composition of the microbiome. The seventeen currently recognized species diverged on the order of the last 1.5 million years across the islands and now occupy a variety of different habitats with dietary diversification^13^. This recently evolved group of species comprise the most intact adaptive radiation of vertebrate species with no known extinctions^14,15^. Ground finches predominantly feed on seeds with the exception of the cactus finch that feeds on flowers and leaf material. Four species are largely arboreal feeders and their diet varies from omnivorous (e.g., small tree finch and large tree finch) to insectivorous (e.g., woodpecker finch and warbler finch)^13,16^. Darwin’s finch populations are well studied on Santa Cruz Island including their phylogeny^17,18^, genetics^19–21^, behavior^22–25^, life history^26^, development^27^, and foraging patterns^16^. The island also has the full range of finch habitats present in the Galápagos archipelago ranging from arid lowlands to humid highlands. The species on Santa Cruz thus provide an excellent model for exploring how well evolutionary and ecological variables explain gut microbiome composition.

A recent study by Michel *et al*.^28^ characterized the gut microbiome of 12 Darwin’s finch species across the archipelago and found a unique gut microbiome community in the vampire finch (*Geospiza septentrionalis*). While they detected no significant impact of finch phylogeny on the microbiome composition, their analysis compared only two species of finches with samples from two different islands. We explore for the first time within-island differences in microbiome across nine Darwin’s finch species sampled in both lowland and highland habitats. We test for effects of host phylogeny, diet, and habitat on Darwin’s finch microbiomes within a single island and breeding season.

## Results and Discussion

The diversity of the gut microbiomes of all nine sympatric species of Darwin’s finches present across highland and lowland habitats on Santa Cruz Island was investigated. We summarized the broad bacterial diversity found in Darwin’s finch samples and test whether they differ by species and habitat. We also examined the signal of co-diversification between host species and their gut microbiomes with Procrustes Analysis of Co-evolution and β-diversity through time. Using stable isotope measurements and foraging observations as dietary proxies, we compared the impact of host phylogeny and diet on the observed variation between microbiomes.

Darwin’s finches were sampled in February 2016 (total N = 63 birds). Three species were sampled in the lowland habitat exclusively: the cactus finch (*G. scandens*), the large ground finch (*G. magnirostris*), and the vegetarian finch (*Platyspiza crassirostris*), while three species were sampled in the highland exclusively: the large tree finch (*Camarhynchus psittacula*), the woodpecker finch (*C. pallidus*), and the warbler finch (*Certhidea olivacea*). Three species were sampled in both highland and lowland habitats: the small ground finch (*G. fuliginosa*), the medium ground finch (*G. fortis*), and the small tree finch (*C. parvulus*) (Table 1).

**Table 1:** Gut microbiome samples from Darwin’s finch species on Santa Cruz island.

Common Name	Abbreviation	Scientific Name	Highland Samples (N)	Lowland Samples (N)	Total per species
Small Ground finch	SGF	*Geospiza fuliginosa*	8	5	13
Medium Ground finch	MGF	*Geospiza fortis*	1	6	7
Large Ground finch	LGF	*Geospiza magnirostris*		8^+^	8
Cactus finch	CF	*Geospiza scandens*		6	6
Small Tree finch	STF	*Camarhynchus parvulus*	9^+^	1^*^	10
Large Tree finch	LTF	*Camarhynchus psittacula*	3		3
Woodpecker finch	WPF	*Camarhynchus pallidus*	5		5
Vegetarian finch	VF	*Platyspiza crassirostris*		4	4
Warbler finch	WF	*Certhidea olivacea*	7		7
Total			33	30	63

^+^No stable isotope samples were collected for 2 STF/Highland and 1 LGF.

*No first foraging observations were collected for STF in the lowland habitat.

Using rRNA gene sequencing on the Illumina MiSeq, 1,671,623 sequences were obtained after quality filtering across all 63 finch fecal samples (mean = 26,367; range = 2,196 to 63,974) with a total of 3,474 amplicon sequence variants (ASVs) (mean = 685, range = 105 to 1,483). Sequence numbers were not significantly different across variables of interest (Table S1) and all following analyses are based on the non-rarefied data for increased statistical power (see Methods – Rarefying reads).

### Taxonomic characterization of Darwin’s finch gut microbiomes

**Figure 1:**
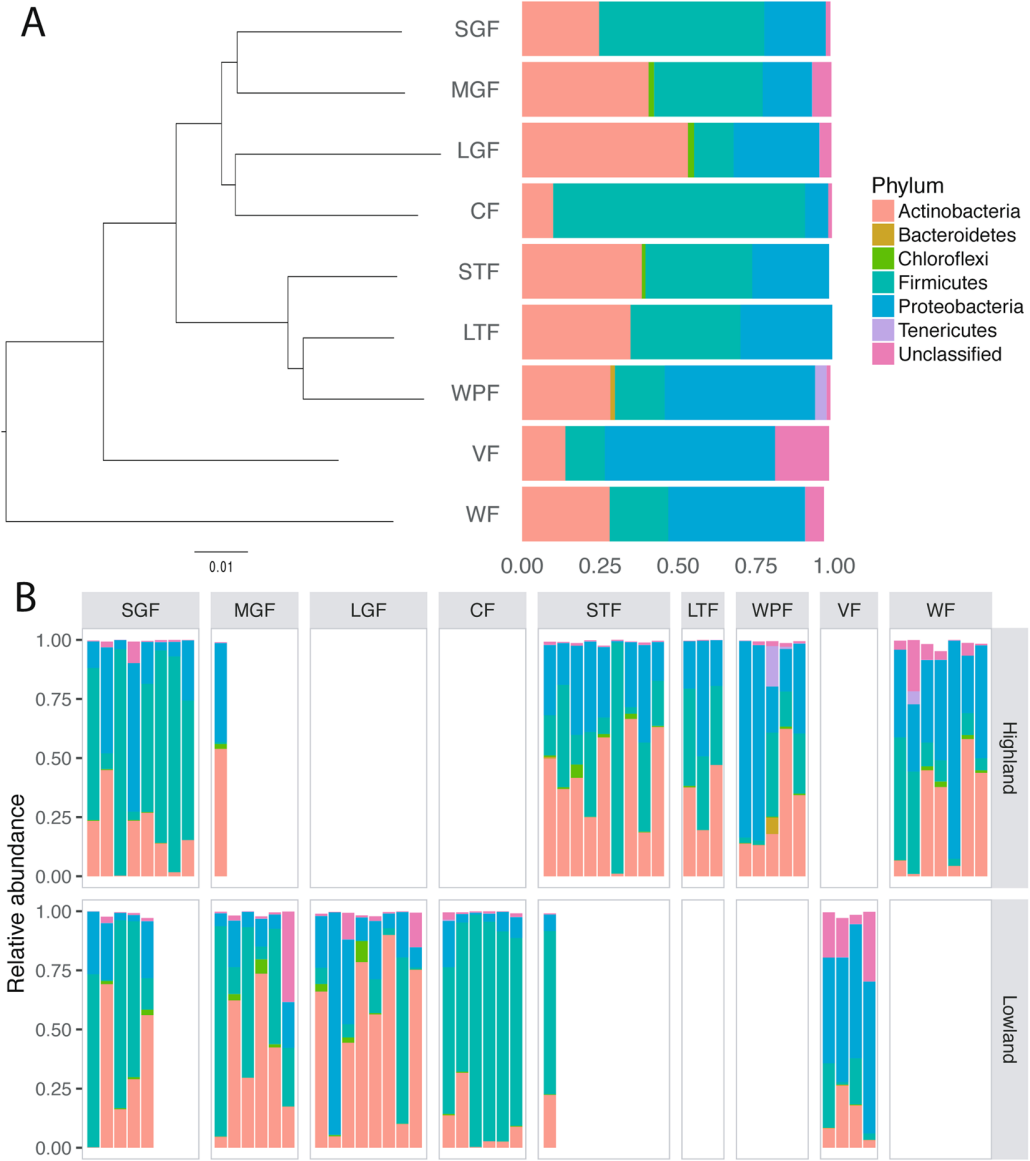
Relative abundance of bacterial phyla in the gut microbiota of Darwin’s finch species. (**A**) Phylogeny of Darwin’s finch species on Santa Cruz island based on whole-genome resequencing^18^ and the mean relative abundance of bacterial phyla across all gut microbiome samples of each species. Species abbreviations in this and all subsequent figures are given in Table 1. (**B**) Relative abundance of the bacterial phyla in individual microbiome samples grouped according to species and habitat. Any bacterial taxa with mean relative abundance within species below 1% was omitted from both plots.

A total of nineteen bacterial phyla were detected in the gut microbiome of Darwin’s finches (Fig. 1, Table S2). The most common bacterial phyla were Firmicutes, Actinobacteria, and Proteobacteria, comprising 28–35% of reads. Unclassified bacterial taxa comprised 4% of the sequences and every other bacterial phyla comprised less than 1% of the sequences. Across host species, the cactus finch and small ground finch had the highest proportion of Firmicutes (82% and 53% respectively), while the large ground finch had the highest proportion of Actinobacteria (56%) (Fig. 1A; Table S3). Based on the visualization of the individual samples grouped by species, there appears to be no clear bacterial phyla solely associated with specific species (Fig. 1B).

The broad taxonomic characterization of the microbiome is consistent with a recent survey of twelve Darwin’s finch species across the archipelago, which noted the same three most common bacterial phyla^28^. Gut microbiome surveys of the broader avian phylogeny found similar patterns with Firmicutes and Proteobacteria as dominant bacterial phyla across bird orders^10^, though Darwin’s finches (order Passeriformes, family Thraupidae) are relatively enriched for the phylum Actinobacteria. The increased abundance of Actinobacteria was also observed in another study on temperate passerine species^11^.

A few genera comprised a significant portion of the total sequences (Fig. S1; Table S4). The genus *Lactobacillus* had the highest relative abundance across all species (26%; Table S4) and in the cactus finch, it comprised the majority of sequences at 77% (Table S5). The most abundant bacterial genera in the other finch species were *Diplorickettsia*, *Rhodospirillum, and Acinetobacter* from the phylum Proteobacteria in the warbler finch (14%), woodpecker finch (16%), and vegetarian finch (17%), respectively, while *Kocuria* from the phylum Actinobacteria was the most abundant genus in the large ground finch (23%). Most of the abundant bacterial genera are soil and plant associated taxa (i.e., *Lactobacillus* (Firmicutes), *Acinetobacter* (Proteobacteria), and *Kocuria* (Actinobacteria)) which likely reflects the large percentage of plant material consumed by the finches. The exception is the bacterial genus *Diplorickettsia*, characterized as obligate intracellular bacteria associated with arthropods^29^ and previously noted to be common within the gut microbiome of temperate bird species^11^. The abundance of arthropod-associated bacterial taxa may be associated with the increased proportion of invertebrates within the warbler finch diet. The two most abundant bacterial genera, *Lactobacillus* and *Acinetobacter*, were also found in the gut microbiomes of the invasive fly^8^, *Philornis downsi*, whose larvae consume the blood and tissue of Darwin’s finches nestlings during development^15^.

The total diversity present in the Darwin’s finch gut microbiome was estimated using three metrics calculated across all samples (mean ± SE): observed ASVs (679.7 ± 41.9), Chao1 (879.5 ± 47.4), and phylogenetic diversity (60.7 ± 2.5). Estimates between species were not significantly different across all alpha diversity measures (Tables S6 and S7). To determine the contribution of shared evolutionary history on the microbial alpha diversity, Pagel’s 𝜆 was calculated for all three metrics using a generalized linear mixed model where host phylogeny and intra-specific variation were both random effects. In all cases, values of Pagel’s 𝜆 were relatively small with large 95% confidence intervals: observed ASVs (0.10; CI: 0 to 0.69), Chao1 (0.10; CI: 0 to 0.68), and phylogenetic diversity (0.14, CI: 0 to 0.74), similar to previous findings from a study in old world passerine species^11^.

### Beta diversity patterns in Darwin’s finch microbiomes

**Figure 2:**
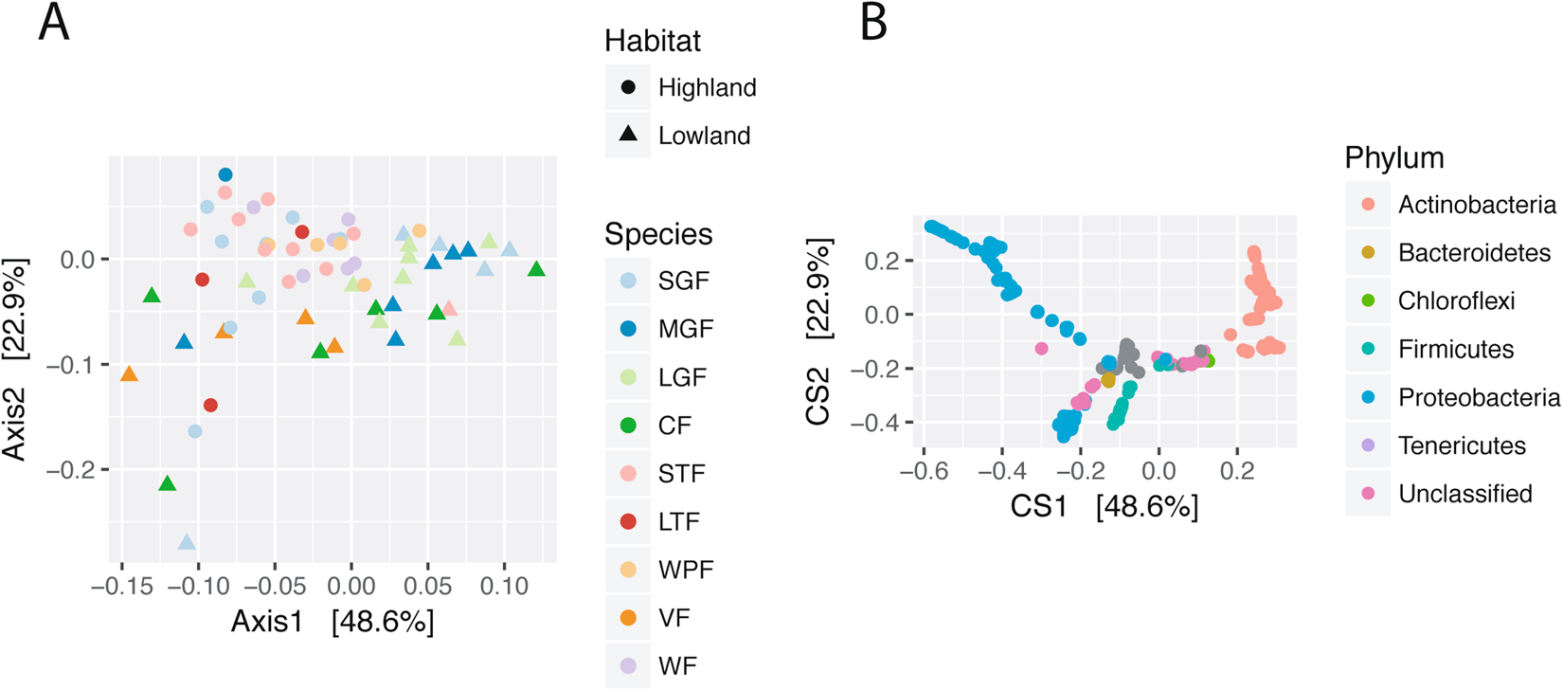
Double principal coordinate analysis (DPCoA) of Darwin’s finch gut microbiome communities. (**A**) Gut microbiome samples are plotted on the first two principal coordinate axes annotated with explained variance, with point color and point shape indicating host species and habitat, respectively. A clear demarcation between highland and lowland samples can be seen along the first axis, with highland samples and lowland samples mostly to the left and right, respectively. (**B**) Individual amplicon sequence variants (ASVs) are plotted in the same ordination space as the gut microbiome samples, with principal coordinate axes annotated with explained variance. Bacterial phyla with at least 1% relative abundance across samples are color-coded; all other ASVs are gray. The demarcation between highland and lowland samples seen in A is recapitulated by the ASVs, with Proteobacteria and Actinobacteria corresponding to highland (left) and lowland (right) samples, respectively.

To visualize differences in the beta diversity of microbiomes across samples, double principal coordinate analysis (DPCoA), a phylogenetic ordination method that allows both the samples and the ASVs to be plotted in the coordinate space, was applied to the log-transformed ASV table. DPCoA demonstrated a demarcation between samples from the highland and lowland habitats, but no clear clustering by species (Fig. 2A). A single warbler finch sample was identified as an outlier and was excluded from further analysis(Fig. S2). Similar results were observed using the weighted UniFrac metric and principal coordinate analysis (Fig. S3). Although almost all finch species have been observed in both habitats^30^, the small ground finch is the most common and widely distributed, enabling multiple samples to be taken from each habitat. Even within the small ground finch, there was a significant separation between highland and lowland samples (PERMANOVA on weighted UniFrac distances p = 0.03). The small ground finch on Santa Cruz is a single, panmictic genetic population^19^, therefore habitat differences are unlikely to be influenced by genetic divergence. Plotting the ASVs in the same ordination space across all finch samples, Actinobacteria and Proteobacteria appear to be associated with the highland and lowland small ground finch samples, respectively (Fig. 2B).

Gut microbiomes differed significantly by both habitat and species as measured by weighted UniFrac distances and calculated with permutational analysis of variance (PERMANOVA) (R^2^ = 0.15 and 0.21, respectively; p = 0.033 for both; Table S8). To determine which species were most dissimilar, *post hoc* ANOVA analysis revealed three pairs of significantly different species: warbler finch vs large ground finch (R^2^ = 0.28; adjusted p = 0.036), vegetarian finch vs small tree finch (R^2^ = 0.31; adjusted p = 0.036), and small tree finch vs large ground finch (R^2^ = 0.22; adjusted p = 0.036; Table S9). All three pairs compare a lowland species to a highland species. Microbiome samples were significantly different in their dispersion depending on habitat (F value = 7.37, p = 0.01), but not depending on species (F value = 1.78, p = 0.10) (Fig. S3). Thus habitat, not species, presents a clear determinant of the gut microbiome in this analysis.

These results are consistent with two recent papers on Darwin’s finch gut microbiomes. First, the lack of gut microbiomes clustering by species recapitulates the findings of Michel *et al*.^28^, who found a distinct microbiome only for the sangivorous vampire finch (*Geospiza septentrionalis*), while the remaining species were indistinguishable from one another. However, we detected differences in habitat, which was not possible in that study given the sampling design. Second, Knutie *et al*.^31^ detected a difference in the gut microbiomes of the small and medium ground finch, specifically along an axis of human presence in their field sites. However, they restricted their sampling to female finches in the lowland habitat. In our study, the samples were collected in field sites without human presence, spanned both highland and lowland habitats, and both sexes, so they are not directly comparable to that dataset. Another possibility for the lack of species clustering in this study is the relatively small sample sizes ranging from 3 to 13 samples per species. The difference in microbiomes between closely related finch species may be much smaller and require higher sample numbers; however, the principal coordinate analyses demonstrate the overlap in microbiome communities even with few samples (Fig. 2A).

### Dietary patterns in Darwin’s finches

**Figure 3:**
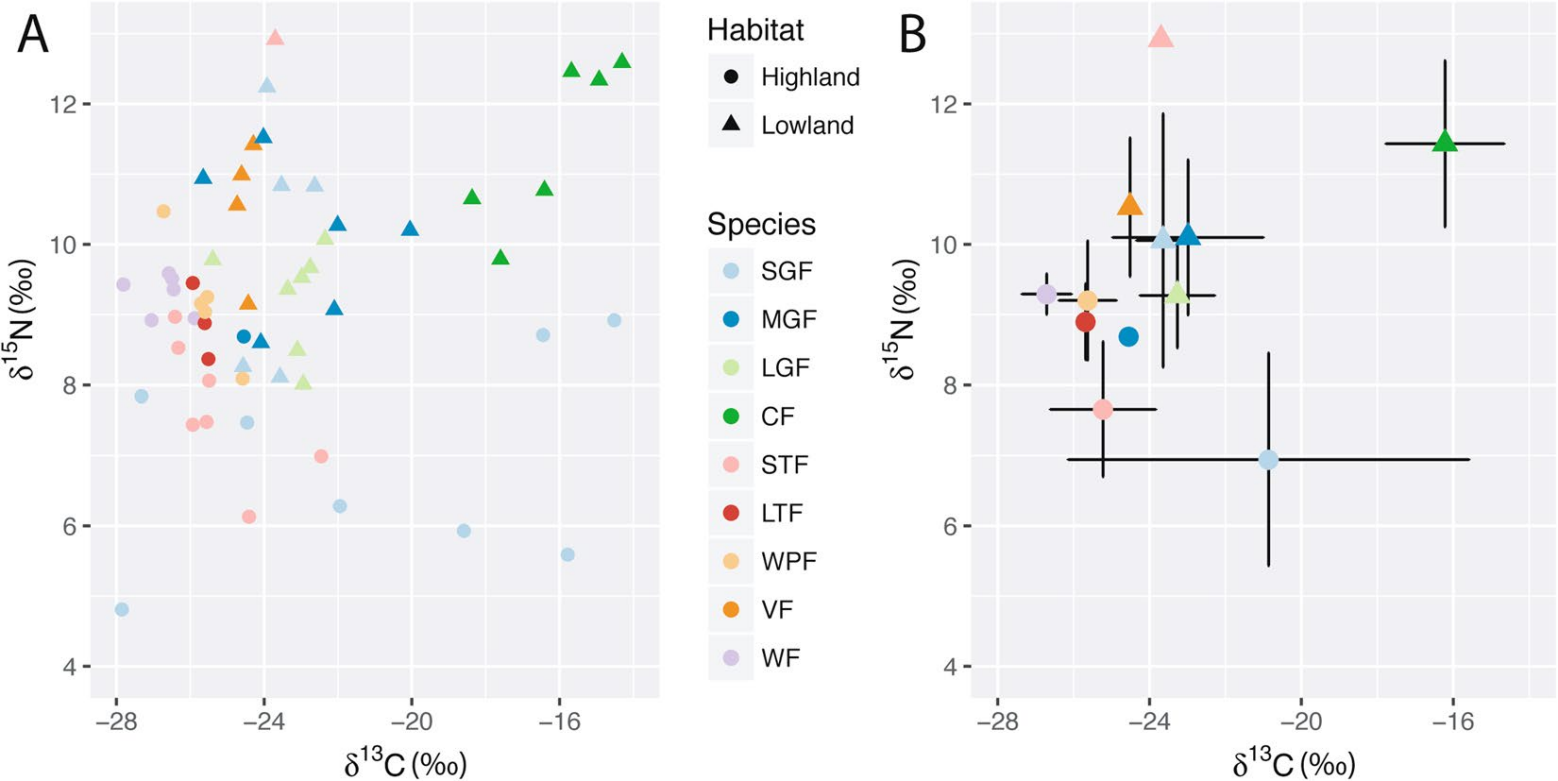
𝛿^13^C and 𝛿^15^N stable isotope measurements for Darwin’s finch species. Point color and point shape indicate host species and habitat, respectively. (**A**) Individual 𝛿^13^C and 𝛿^15^N values for each finch with gut microbiome samples. (**B**) Mean 𝛿^13^C and 𝛿^15^N values for each species and habitat with standard deviation. The small ground finch (SGF) is the only species with multiple samples per habitat and has distinct 𝛿^13^C and 𝛿^15^N values dependent on the habitat of origin.

To place the characterized microbiomes in an ecological context, we estimated dietary differences using two methods. First, stable isotope ratios (𝛿^13^C and 𝛿 ^15^N) were determined using blood samples collected from each finch used for microbiome analyses (N = 60). Carbon isotope values for the tree finches (mean 𝛿^13^C = −25 to −26.7‰) were less enriched than those for the ground finches (mean 𝛿^13^C = −21.9 to −24.5‰) with the cactus finch having the most enriched signal (mean 𝛿^13^C = −16.2‰) (Fig. 3; Table S10). The carbon isotope values differed significantly by both habitat and species (p = 5.5e-05 and 2.9e-05, respectively; Table S11). Post hoc analysis revealed the cactus finch to drive the species difference observed, with its carbon isotope values significantly different compared to all four tree finch species (Table S12). This signal is consistent with the cactus finch feeding on *Opuntia* cacti species that fix carbon via the Crassulacean acid metabolism (CAM) pathway^32^. Nitrogen isotope (𝛿^15^N) measurements showed a weaker, but still significant difference between habitat and species (p = 1.6e-05 and 0.003, respectively, Table S11). Again the cactus finch exhibited the most enriched stable isotope value (𝛿^15^N = 11.4‰) and was significantly different in comparison to the small ground finch and small tree finch (Table S13). The mean values of all other finch species ranged from 𝛿^15^N 8.1‰ to 10.5‰. Stable isotope values also showed a distinction by habitat of origin. Lowland samples generally had lighter carbon isotope values and heavier nitrogen isotope values, even within species such as the small ground finch (Fig. 3B). However, within the small ground finch, 𝛿^15^N was significantly different (p = 0.02) while 𝛿^13^C was not (p = 0.38). These stable isotope patterns corroborate the microbiome distinction seen across habitats.

Next, we collected first foraging observations to characterize broad dietary patterns in each species and habitat. These observations showed differing proportions of food type dependent on species (N = 201 observations, N per species ranging from 6 to 41; Table S14; Fig. S4). In the lowlands, the large ground, cactus, and vegetarian finches were observed to consume plant material including seeds, flowers, and leaves. The small ground and medium ground finches consumed a proportion of invertebrates (33% and 5%, respectively) in addition to these plant sources. In the highlands, the woodpecker and warbler finches were only observed consuming invertebrates, while the small tree and large tree finches included some plant material (10% and 20%, respectively), congruent with previous surveys^16^. The diet of the small ground finch was similar across both habitats, apart from an exchange of flowers over fruit in the lowland and highland, respectively, similar to previous surveys^33,34^. Though some species had relatively few foraging observations, as they were consistent with previous studies and diet can shift the microbiome rapidly, we prioritized the data collected in the same field season as the gut microbiome samples for downstream analyses. Diet can shift according to habitat, as shown in the medium ground finch, which consumed more invertebrates in the highlands and more flowers and leaf material in the lowlands.

Using both stable isotopes and foraging observations, we characterized the diets of Darwin’s finches on Santa Cruz. The stable isotope values showed a separation by habitat but not by species, with the exception of the heavier 𝛿^13^C values in the cactus finch. Even within the small ground finch that was observed to consume similar proportions of food items, the 𝛿^15^N values were enriched in the lowland samples, thus these values are unlikely to reflect trophic partitioning^35^. Visual comparison of the foraging and microbiome data (Fig. S5) shows clearly that habitat and diet are interconnected, with highland species consuming more invertebrates and lowland species consuming more plant material. A beta regression confirmed that the proportion of plant material consumed was significantly different across habitats, with less consumed in the highland (estimate = −3.2, p = 4.0e-04).

Dietary shifts across seasons are known to impact the gut microbiome of organisms, and in Darwin’s finches, foraging patterns change significantly between the wet and dry seasons^13,16^. Food scarcity in Darwin’s finches during drought years on the Galápagos has driven the most impressive examples of observed natural selection^36^. It is possible our data, collected during the wet season, demonstrate the breadth of microbial diversity in the Darwin’s finch species but do not reflect the restricted dietary niches that facilitated speciation events that are evident during the dry season. Indeed, a previous comparison of the gut microbiome in Darwin’s finch species found significant differences between seasons within the small and medium ground finch^28^.

### Comparative analyses of microbiomes with host phylogeny and diet

#### Testing co-phylogeny of Darwin’s finches and their microbiomes

Co-diversification between host species and their gut microbiome communities has been investigated across the animal kingdom and at multiple scales^37–39^. To test congruence between the phylogeny of Darwin’s finches and their gut microbiomes, the correlation between the weighted UniFrac distances of the microbiome samples and the genetic distances of the host species was tested using the Procrustean approach to co-phylogeny (PACo)^40^. PACo analysis showed significant correlation between the host phylogeny and the microbiome (R^2^ = 0.18, p < 0.001) (Table S15). PACo analysis was also applied to the stable isotope and foraging data. Foraging data had a similar procrustean correlation coefficient (R^2^ = 0.18, p < 0.001) while stable isotope values were lower (R^2^ = 0.06, p = 0.013).

The moderate correlation between microbiomes and host phylogeny demonstrate an effect of the evolutionary relationship among host species on the composition of the gut community. In the context of previous studies on host phylogeny and bird microbiomes, these correlation coefficients are about half the correlation coefficient previously described across 51 passerine species, where R^2^ = 0.35^11^, which are congruent with the much larger portion of the avian phylogeny and therefore longer timescale of diversification encompassed by that study. The significant correlation between the Darwin’s finch phylogeny and the microbiomes is based on all nine species on Santa Cruz and leverages the phylogenetic distances between finch species, whereas Michel *et al*.^28^ used two species as categorical variables in their test which found no correlation.

The foraging data had a similar correlation coefficient indicating an analogous effect of diet. The similar coefficient values shown with PACo show the need for additional analyses to disentangle the independent effects of phylogeny and diet.

#### Investigating time scales of correlation between host and microbiomes

To test whether the congruence found with PACo for both foraging data and host phylogeny fit with a model of co-diversification, beta diversity through time analysis (BDTT)^9^ was applied to the microbiome data. BDTT analysis samples the bacterial phylogeny at given time intervals, providing a correlation profile between the bacterial taxa and corresponding metadata at different phylogenetic resolutions. Profiles that show high correlation coefficients further back in evolutionary time indicate that more ancient bacterial lineages are driving the observed correlation. In contrast, profiles with high correlation coefficients at more recent dates but low correlation further back in time signify that recent bacterial diversification is responsible for the similarity between the microbiome and metadata. Importantly, since this analytic technique samples the bacterial evolutionary timescale, the correlation profiles extend far beyond the diversification of Darwin’s finches themselves (~1 mya). BDTT specifically assesses whether more recent or more ancient bacterial evolution explains the correlation seen. If it is more recent bacterial diversification, this implies co-diversification with the host. In contrast, if it is more ancient bacterial lineages, this implies horizontal or environmental filtering explains the gut microbiome diversity observed.

**Figure 4:**
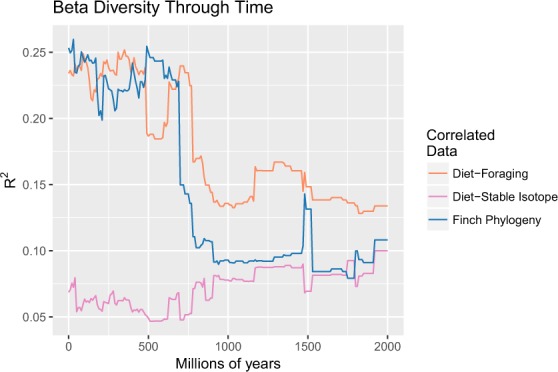
Beta diversity through time applied on the gut microbiome data. Lines show pairwise Sorensen dissimilarities of Darwin’s finch gut microbiome samples determined by time slices every 10 Mya correlated to pairwise dietary distances calculated with first foraging observations (red), pairwise dietary distances using 𝛿^13^C and 𝛿^15^N stable isotope measurements (green), and pairwise host phylogenetic distances (blue).

Finch phylogenetic relationships and foraging data correlated most strongly with the beta diversity distance, though at slightly different time scales. For both variables, the correlation coefficient remained below 0.15 until 2000 mya, indicating a lack of sufficient bacterial diversity prior to this time point (Fig.S6). The finch phylogeny and foraging data both increased past R^2^ = 0.20 around 750 mya (finch phylogeny: 690 mya, R^2^ = 0.23, p = 0.003; foraging data: 770 mya, R^2^ = 0.22, p = 0.007) and remain between R^2^ = 0.20 and R^2^ = 0.25 until present, with the maximums occurring at 30 mya (R^2^ = 0.26, p = 0.001) and 350 mya (R^2^ = 0.25, p = 0.002), respectively (Fig. 4). The maintenance of the correlation well beyond the divergence time of Darwin finch species (~1 mya) indicates that the present correlation between finch phylogeny and microbiome is due to divergence among ancient bacterial lineages instead of recent bacterial evolution in conjunction with host speciation. In other words, the diversity of bacteria observed in the finch gut are most likely due to environmental filtering via the different diets as opposed to specific bacterial lineages co-diversifying with the species. The correlation coefficient between beta diversity distance and stable isotope values was less than 0.10 across all time points (peak at 1920 mya; R^2^ = 0.10; p = 0.18), indicating that the stable isotopes do not explain the gut microbiome diversity observed.

This similarity in correlation profile between the host phylogeny and foraging data contrasts with the pattern seen when BDTT was applied to a dataset of 33 mammalian species^9^. There the correlation with host phylogeny peaked around 100 mya, near the estimated time of divergence for the mammalian host species and then dropped off significantly while the correlation with diet peaked around 500 mya but was not significant at present. Though it is possible the divergence between Darwin’s finch species is simply too recent for BDTT to disentangle, the similarity in correlation profile between host phylogeny and foraging data are consistent with dietary preferences mainly driving the differences observed between microbiome samples as opposed to evolutionary diversification processes during the Darwin’s finch adaptive radiation.

#### Comparing microbiome variance explained by host phylogeny and diet

Given the similarity in correlation with PACo and BDTT, we used variation partitioning to compare the amount of variance in the microbiome explained by the finch phylogeny, stable isotope values, and foraging data. Total explained variance with all three explanatory tables had an adjusted R^2^ = 0.16 (Fig. S7). Foraging data had the highest correlation with the microbiome (adjusted R^2^ = 0.175, p = 0.001) while the finch phylogeny (adjusted R^2^ = 0.066, p = 0.010) and stable isotope values (adjusted R^2^ = 0.072, p = 0.009) showed a weaker correlation and were comparable in their explained variance. The total explained variance was lower than the sum of the individual explanatory tables due to the overlapping nature of the partitions. After controlling for variation explained by the overlap between explanatory tables, only foraging data uniquely explained variation in the microbiome (adjusted R^2^ = 0.053, p = 0.014). The generalist trend in dietary patterns observed in Darwin’s finch species suggests that their microbiome has greater variability in comparison to the tight associations observed between microbiome communities and their herbivorous or carnivorous mammalian hosts^9^.

### Random forest classification of finch habitat based on gut microbiome communities

To determine how well the gut microbiome community can distinguish between habitats and host species, a random forest algorithm was applied to the samples, which creates a classification model using the log transformed abundance of each ASV across samples^41,42^. Classification of the gut microbiome samples by habitat was significantly higher than the no information rate with only two highland samples misclassified as having lowland origin (total samples: 57, accuracy = 0.96, no information rate = 0.55, p < 0.0001). Conversely, classifying samples by host species was unsuccessful, with the accuracy of the model equal to the no information rate (accuracy = 0.23, p = 0.55) (Fig. S8), corroborating the lack of clear species clusters in the DPCoA visualization (Fig. 2). This suggests the lack of species-specific taxa in the gut microbiomes of Darwin’s finches.

To determine which bacterial taxa are associated with each habitat, we used the ranked importance for classification of each ASV. The top 10 ASVs for habitat classification ranked by the mean decrease in accuracy across all 57 models are shown in Table S16. In concordance with the DPCoA plot, these ASVs are all in the bacterial phyla Actinobacteria or Proteobacteria. Plotting the abundance of the most important ASV (phylum Actinobacteria, genus *Tetrasphaera*) shows its presence in most lowland samples and absence in most highland samples (Fig. S9). The lack of species-specific bacterial taxa in Darwin’s finches supports a model of microbiome assembly that is more dependent on locality than host species identification, which is consistent with our PACo and BDTT analyses, as well as findings from a separate study on brood-parasitic cowbird where geography explained the most variation in the gut microbiome^43^.

## Conclusion

The combination of a well-studied host phylogeny, individual stable isotope values, and foraging data specific to a single breeding season enabled us to identify significant effects of diet and phylogeny on the composition of the gut microbiome in Darwin’s finches. However, two findings suggest that host phylogeny is not the primary driver of community composition. First, the correlation profile from BDTT analyses for the host phylogeny extends well back in the bacterial phylogeny, which is consistent with environmental filtering as opposed to co-speciation of bacterial lineages within each Darwin’s finch species. Second, variation partitioning was able to quantify a unique contribution of foraging data to the observed correlation with the microbiomes, which is in contrast to the host phylogeny that co-varied with other variables. Adaptive radiations, such as the Darwin’s finch system, provide a powerful evolutionary lens to explore the role of ecological niches for speciation and as this study shows, they can also illuminate the role of ecological variables for determining a hosts’ microbiome.

## Materials and Methods

### Ethics statement

All samples were collected with permission from the Parque Nacional Galápagos and Ministerio del Ambiente, Ecuador (Research permit No. PC-23–16). All collection protocols were approved by the Institutional Animal Care and Use Committee in the Faculty of Arts and Sciences at Harvard University (Protocol 15-08-249). All methods were performed in accordance with the relevant guidelines and regulations.

### Study sites and species

Fieldwork was conducted in February 2016 on Santa Cruz Island, Galápagos Archipelago, Ecuador. Sampling sites were located in both highland (0°37′S, 90°23′W) and lowland (0°40′S, 90°13′W) habitats. All nine extant finch species on Santa Cruz were sampled and the number of samples per species per habitat are detailed in Table 1. Three species were only sampled in the lowlands: the cactus finch (*Geospiza scandens*; n = 6), the large ground finch (*Geospiza magnirostris*; n = 8), and the vegetarian finch (*Platyspiza crassirostris*; n = 4) and three species were only sampled in the highlands: the large tree finch (*Camarhynchus psittacula*; n = 3), the woodpecker finch (*Camarhynchus pallidus*; n = 5), and the warbler finch (*Certhidea olivacea*; n = 7). Two species had a single sample in the second habitat - the medium ground finch (*Geospiza fortis*; n = 6 lowland (L)/1 highland (H)) and the small tree finch (*Camarhynchus parvulus*; n = 1L/9H) - while the small ground finch (*Geospiza fuliginosa*) was the only species with multiple samples in both habitats (n = 5L/8H).

### Sample collection

Finches were caught using mist nets and tagged with an aluminum ring imprinted with a unique identifier. Eight morphological measurements were taken for all finches sampled^44^. These included (1) beak-head (beak tip to back of head), (2) beak-naris (beak tip to anterior end of the naris), (3) beak-feather (tip of beak to feather line), (4) beak depth (at the base of the beak), (5) beak width (at the base of the beak), (6) naris diameter (taken from extremes of naris opening), (7) tarsus length, (8) wing length, and (9) body mass. Dial calipers were used to take morphological measurements to the nearest 0.01 mm and Telinga electronic scales were used to measure mass to the nearest 0.01 g. Individuals were classified into species using the morphological measurements and established protocols^13,44,45^. Blood samples (10 μl) were collected for stable isotope analyses, dried on small pieces (roughly 0.5 × 0.5 cm^2^) of quartz fiber filter paper (Schleicher and Shuell, Dassel, DE), and stored in microcentrifuge tubes with a silica gel bead as desiccant at room temperature.

After morphological measurements and blood sample collection, fecal samples were collected by placing each finch into a 7″ × 7″ × 7″ cage lined with UV-sterilized parchment paper. Cages were covered with fabric and finches were monitored until defecation for a maximum of 30 min before release. Feces were immediately transferred from the parchment paper with bleach cleaned spatulas into pre-weighed microcentrifuge tubes containing 1 ml of DNA/RNA Shield (Zymo Research, Irvine, CA) and mixed by shaking the tubes by hand before storage at −20 °C within 4 hours of collection to prolong the longevity of the DNA stabilization buffer. Fecal samples were shipped at room temperature and stored at −80 °C in the lab until further analysis.

### Foraging observations

To quantify the diet patterns across species in both habitats, first foraging observations were collected from highland and lowland sampling sites^33,46^. Such observations have been shown to accurately measure the pattern of food items consumed among Darwin’s finches^16,34,47^. At each site, a single walk through of 1 hour was conducted with no overlaps or doubling back to avoid observing the same individuals. During the walkthrough, individual finches were observed until the first food item was ingested. The food item consumed was recorded as one of five categories: invertebrate, seed, flower, leaf, or fruit. Due to the tame nature of Darwin’s finches, the majority of observations were made within 10 m of the focal individual.

### Fecal DNA Extraction and 16S rRNA gene sequencing

DNA was extracted from feces in the laboratory using the ZR Fecal Miniprep kit (Zymo Research, Irvine, CA) following manufacturer’s instructions with the following changes. To minimize loss of biological material, BashingBeads (Zymo Research, Irvine, CA) were added directly to the collection tubes with the fecal sample in DNA/RNA Shield, which acted as the lysis buffer. Samples were homogenized in a FastPrep FP120 (Qbiogene, Carlsbad, CA) for six rounds of 45 s at speed 6.5 m/s. Between each round, tubes were cooled on ice for 3 min. All liquid transfer steps were performed in a laminar flow hood to minimize environmental contamination.

The V4 region of the 16S rRNA gene was amplified using NEBNext Q5 HotStart HiFi MasterMix 2× (New England Biolabs, Ipswich, MA) and previously designed dual-index barcoded universal primers^48^. For each fecal DNA sample, triplicate 25 μl PCR reactions were performed containing 12.5 μl master mix, 9.5 μl molecular grade water, 0.5 μl of 10 M stock for each primer, and 2 μl of DNA template. PCR conditions consisted of initial denaturation at 94 °C for 5 min followed by 20 cycles of 98 °C for 20 s, 55 °C for 15 s, 72 °C for 40 s, and a final extension at 72 °C for 5 min.

All PCR products were purified using 0.66X Aline PCRClean DX (Aline Biosciences, Woburn, MA) to size select for the ~450 bp PCR product. Purified PCR products were visualized and quantified using High Sensitivity D1000 ScreenTape on an Agilent 2200 TapeStation (Agilent, Santa Clara, CA) and pooled in equimolar concentrations for sequencing on a single MiSeq run (Illumina, USA) using v2 chemistry and 2 × 250-bp paired-end reads at the Harvard Biopolymers Facility (Boston, MA).

### Contamination controls

Given the low DNA content of bird feces^49^, we were concerned about the influence of environmental microbial contamination in analyzing the sequences^50^. To understand the sources of contamination, controls were included at both steps of sample processing: DNA extraction and amplification. To evaluate contaminants from the DNA extraction kits, for each kit we included a mock community of bacterial cells using 75 μl of ZymoBIOMICS Microbial Community Standard (Zymo Research, Irvine, CA) and a no sample extraction control with only DNA/RNA Shield. To assess contaminants from PCR amplification reagents, for each 96-well plate of PCR amplification, a mock community of bacterial DNA was amplified in triplicate with 2 μl of a 1:10 dilution of ZymoBIOMICS Microbial Community DNA Standard (Zymo Research, Irvine, CA) and a triplicate no template control reaction. Greater than 99.8% of all reads from ZymoBIOMICS Microbial Community standards and DNA standards mapped to the expected genera. None of the extraction or no template controls produced quantifiable PCR product and were excluded from further analysis.

### Sequence processing

Sequences were demultiplexed according to the dual-index barcodes by the Harvard Biopolymers Facility (Boston, MA) and all the following sequence processing steps were performed in R version 3.4.0^51^. The fastq files for each sample were converted into amplicon sequence variants (ASVs) using DADA2 with parameters as described in^52^. ASVs were taxonomically classified with the RDP v14 training set^53^ and chimeras were removed as implemented in DADA2. After initial processing a total of 1,865,835 reads and 6,225 ASVs were identified across all samples.

### Sequence filtering

The following steps were taken to produce the final dataset for analysis. To remove likely environmental DNA contaminant sequences, the frequency based *decontam* algorithm^54^ was applied to the dataset, which removed 26,484 reads (1.42%) and 122 ASVs (1.96%). To reduce the influence of ASVs present in only a few samples, a 5% prevalence filter was applied, which removed 57,335 (3.12%) and 2,570 ASVs (42.11%). After taxonomic assignment, any sequences not classified as Bacteria were removed, subtracting 2,518 reads (0.14%) and 23 ASVs (0.65%). Finally, sequences classified as chloroplasts were removed, subtracting 107,875 reads (6.06%) and 36 ASVs (1.03%). The final dataset included 1,671,623 reads and 3474 ASVs.

### Rarefying reads

To ensure sample library sizes were not driving the patterns observed in the data, the following categorical variables were checked for significant differences in mean library size using the Kruskal-Wallis rank sum test and library size distribution using Levene’s test as implemented in the R package car^55^: species, habitat, sex, and PCR plate. None of the variables were significantly different in mean library size or library size distribution (Table S1). Therefore, for increased statistical power in detecting differences between microbiome samples, all following analyses were performed with non-rarefied microbiome data^56^.

### Alpha diversity analyses

To calculate the relative abundance of bacterial phyla present in the gut microbiome of each Darwin’s finch species, reads were transformed to proportions by sample and then averaged across all microbiome samples per finch species. For species Darwi estimates, observed ASVs and Chao1 estimates were calculated in the R package phyloseq^57^ while phylogenetic diversity was calculated with the R package picante^58^. The Chao1 estimate uses information on the frequency of rare species to estimate the total number of species in the assemblage, including undetected species^59^. Phylogenetic diversity sums the total branch length of the resulting bacterial phylogeny^60^. To check for phylogenetic signal of the alpha diversity, Pagel’s lambda was calculated using a Markov-chain Monte Carlo generalized linear mixed-effects model as implemented in the R package MCMCglmm^61^. The phylogenetic signal was calculated as a random effect, incorporating both variation between species as well as within species between the multiple measurements. The model was run with 5,000,000 iterations, a 10,000 step burn in, and 500 step thinning intervals.

### Beta diversity analyses

To visualize differences between microbiome samples, double principal coordinate analysis (DPCoA) was applied to the log-transformed ASV table as implemented in the R package phyloseq^57^. DPCoA is a dissimilarity metric which incorporates both quantitative and phylogenetic information about the microbiome samples^62^. To assess the differences in community composition of the gut microbiomes between samples, weighted UniFrac distances^63^ were calculated between all samples. All abundance data were log transformed prior to distance calculations as an approximate variance stabilization method. To check for the homogeneity of the multivariate dispersions of the distance metrics, the *betadisper* function was used as implemented in the R package vegan^64^. To test the significance of categorical variables, permutational analysis of variance (PERMANOVA) was used as implemented in the R package vegan function *adonis*^64^. The statistical tests chosen here are non-parametric and permutation based, making them suitable to analyze and interpret the patterns observed even with a smaller number of samples.

### Stable isotope analysis

To assess differences in diet between the finches sampled, stable isotope analysis was performed using blood samples dried on quartz fiber filter paper. These were packaged in 5 × 9 mm tin capsules for analysis (041077, Costech Analytical Technologies, Inc, Valencia, CA). Values are expressed in ‰ as 𝛿^13^C = [(R_sample_/R_std_) − 1], where R_sample_ = ^13^C/^12^C in a sample, and R_std_ = ^13^C/^12^C in the Vienna Pee Dee Belemnite standard. Similarly, 𝛿 ^15^N = [(R_sample_/R_std_) − 1], where R_sample_ = ^15^N/^14^N in a sample, and R_std_ = ^15^N/^14^N in atmosphere. 𝛿^13^C and 𝛿^15^N values were measured on a Thermo Scientific Delta V paired with a Costech 4010 elemental analyzer and a high-temperature conversion elemental analyzer at the Center for Stable Isotopes at the University of New Mexico (Albuquerque, NM). A known protein standard was run at multiple concentrations as a control. 𝛿^13^C values were adjusted by the mean difference between the measured values for the protein and the known value (−1.64‰). 𝛿^15^N values for samples below 1000 mV were error-corrected using a linear regression on the protein standard (R^2^ = 0.77).

Differences in stable isotope ratios by habitat and species were tested using Kruskal-Wallis test in R. Post hoc pairwise Dunn tests between species were performed using the dunnTest function in the R package FSA.

Two small tree finches and one large ground finch did not have blood samples collected and were excluded from all analyses using stable isotope ratios.

PACo analysis was also used to calculate the correlation between stable isotope values and microbiome community composition. Euclidean distance matrices were calculated between the stable isotope signatures of each sample and between the mean values of stable isotopes in each species and each habitat combination. The stable isotope distance matrices were used in PACo analyses of the microbiome weighted UniFrac beta diversity metric.

### Foraging data analysis

To summarize the foraging data, the food items seed, flower, leaf, and fruit were combined into the category plant. We calculated the proportion of plant versus insect food items to summarize the general observed dietary patterns for each finch species in both habitats. Since no foraging observations were made of small tree finches in the lowlands, the single small tree finch fecal and stable isotope samples from the lowlands were excluded from any analysis using foraging data. Proportions of plant food consumed were tested using beta regression with the function *betareg* in the R package betareg.

For PACo analyses and analysis of beta diversity through time (see below), Euclidean distances were calculated between the proportion of observations across the five food items. For variation partitioning, principal component analysis was applied to the proportion data using the function *rda* as implemented in the R package vegan^64^.

### Procrustean Approach to Cophylogeny (PACo)

To assess congruence between the phylogenetic diversification of Darwin’s finches and their gut microbiomes, the Procrustean Approach to Cophylogeny (PACo)^40^ was applied to the data. PACo was designed to detect the similarity of evolutionary patterns in host-parasite associations. Here, the microbiome samples are treated as the ‘parasites’ to compare with the host species genetic distances. Darwin’s finch species’ genetic distances for the PACo analysis were based on whole genome resequencing encompassing more than 44 million variable sites with representative individuals chosen from Santa Cruz when available (Lamichhaney, personal communication^18^. Microbiome distances were calculated using the weighted UniFrac metric^63^, to produce a quantitative distance comparison that incorporated the phylogeny of the microbial community. PACo analysis was run as implemented in the R package paco^65^, with 10,000 permutations to test the significance of the signal. Using the symmetric calculation, the correlation coefficient r was calculated as r = (1-ss).

As three samples lacked stable isotope data and one sample lacked foraging data, a total of four samples were excluded from PACo analysis with the finch phylogeny (see below).

### Beta diversity through time (BDTT) analysis

To further disentangle the contribution of host phylogeny and diet to the microbiome composition, the beta diversity through time (BDTT) metric^9^ was applied to the dataset. Only species and habitat combinations with multiple samples were included in the analysis, which removed the medium ground finch highland sample. Only the small ground finch had multiple samples in both habitats and therefore was split into highland and lowland microbiome profiles. The mean relative abundance of ASVs was calculated across all samples of each species and habitat combination. BDTT was calculated as described in Groussin *et al*.^9^. Briefly, the ASV sequences were aligned using the SINA algorithm^66^ and all sites with more than 95% gaps were removed. The bacterial phylogenetic tree was calculated with FastTree^67^ using the GTR model and default CAT approximation to model rate heterogeneity across sites and with the constraint that all bacterial phyla and all classes within Proteobacteria were monophyletic. The root of the tree was placed between Actinobacteria and the rest of the taxa. PATHd8^68^ was used to time-calibrate the tree with a maximum age of 3.8 Gya for the root as an estimate of the earliest signs of life^69^. The time calibrated bacterial phylogeny was sliced in units of 10 Mya and Spearman correlations of Bray-Curtis dissimilarity between species were calculated against three distance matrices using Mantel tests: host phylogenetic distance, Euclidean distance between the mean stable isotope values, and Euclidean distance between foraging data across the five food items.

### Variation partitioning

To compare the amount of variation explained by host genetic distance, stable isotope values, and diet distance as calculated from first foraging observations, variation partitioning by redundancy analysis^70^ was used as implemented with the *varpart* function in the R package vegan^64^. The microbiome distance matrix was used as a response variable with three explanatory tables: the first two principal coordinate axes of the host genetic distance, the 𝛿^13^C and 𝛿^15^N stable isotope values, and the first two principal component axes of the first foraging observations. Significance of the distance based redundancy analysis was assessed using the *anova.cca* function implemented in vegan.

### Random forest analysis

To determine whether the gut microbiome communities could differentiate between categorical variables of interest, a random forest classifier^41^ was applied to the ASV table as implemented in the R package randomForest^71^, using leave-one-out cross validation. ASVs with the top importance for classification were determined by calculating the mean decrease in accuracy across all models. Accuracy was defined by the number of samples correctly classified based on the category of interest, which was calculated along with confusion matrices in the R package Caret^72^.

## Supplementary information

Supplementary Figures Loo et al. Host phylogeny, diet, and habitat differentiate the gut microbiomes of Darwin’s finches on Santa Cruz Island

## Data Availability

All fastq files have been deposited in the NCBI sequence read archive, accession number PRJNA523677.
