# Supplementary material for: Host phylogeny, diet, and habitat differentiate the gut microbiomes of Darwin’s finches on Santa Cruz Island
Source: Sci Rep. 2019 Dec 11;9:18781. doi: 10.1038/s41598-019-54869-6 (PMC6906294; doi:10.1038/s41598-019-54869-6)
Supplement: Supplementary file 1 — Supplementary Figures Loo et al. Host phylogeny, diet, and habitat differentiate the gut microbiomes of Darwin’s finches on Santa Cruz Island [file 41598_2019_54869_MOESM1_ESM.docx]

Supplementary Material

Host phylogeny, diet, and habitat differentiate the gut microbiomes of Darwin’s finches

Wesley T. Loo^1^, Jefferson García-Loor^2^, Rachael Y. Dudaniec^3^, Sonia Kleindorfer^4,5*^, Colleen M. Cavanaugh^1*^

^1^Department of Organismic and Evolutionary Biology, Harvard University, Cambridge, MA, USA

^2^ Laboratory of Evolutionary Biology, San Francisco University, Quito, Ecuador

^3^Department of Biological Sciences, Macquarie University, Sydney, NSW, Australia

^4^College of Science and Engineering, Flinders University, Adelaide, SA, Australia

^5^Konrad Lorenz Research Center for Behaviour and Cognition and Department of Behavioural Biology, University of Vienna, Vienna, Austria

* Address correspondence to [sonia.kleindorfer@flinders.edu.au](mailto:sonia.kleindorfer@flinders.edu.au) or [cavanaug@fas.harvard.edu](mailto:cavanaug@fas.harvard.edu)

Supplementary Figures

Figure S1. Mean relative abundance of bacterial taxa grouped at varying taxonomic levels.

Relative abundance was calculated for each bacterial ribosomal sequence variant (ASV) according to the total number of sequences per sample. Only taxa with mean relative abundance above 5% were included. Mean relative abundance for ASVs grouped for bacterial order (A) and genera (B). Note that taxa labeled as ‘Unclassified’ at these taxonomic levels may be classified at larger taxonomic levels.

Figure S2. Double principal coordinate analysis with all samples showing the outlier warbler finch sample.

This sample was excluded from further analysis.

Figure S3. Beta dispersion of weighted UniFrac distances of gut microbiome samples categorized by habitat and finch species.

Weighted UniFrac distances were plotted on the first two principal coordinate axes and colored by category. Habitat displayed a significant difference in dispersion while species did not.

Figure S4 First foraging observations of Darwin’s finch species across both habitats.

Two species, the small ground finch (SGF) and medium ground finch (MGF) occur in both habitats.


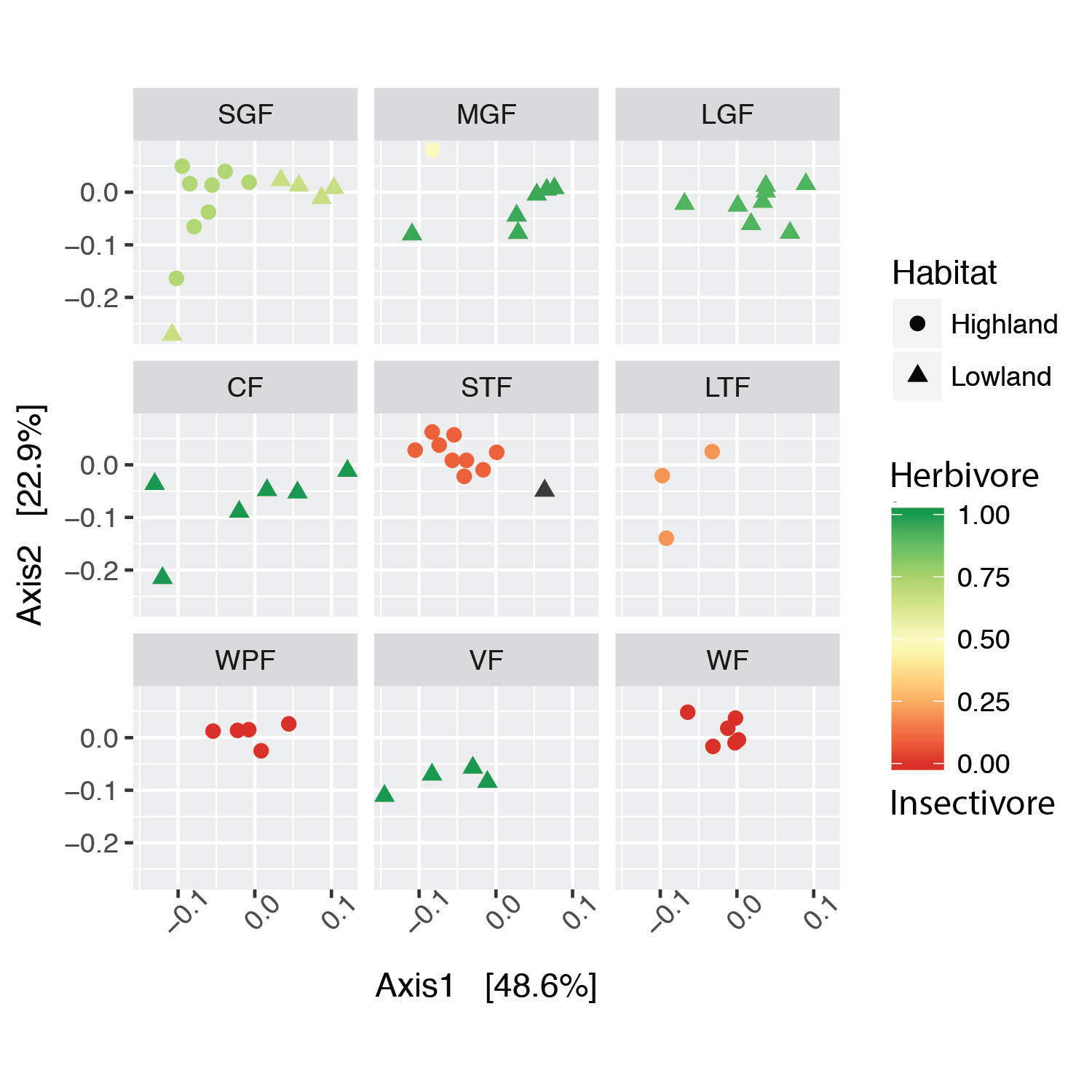


Figure S5. Double principal coordinate analysis (DPCoA) of Darwin’s finch gut microbiome samples overlaid with foraging data.

Darwin’s finch gut microbiome samples are plotted with DPCoA ordination and facetted by finch species. The color of each point corresponds to the proportion of the diet coming from plants vs invertebrates as estimated by foraging observations. The lowland small tree finch is colored gray as no foraging observations were collected for that species and habitat combination.

Figure S6. Beta Diversity Through Time with full bacterial timescale.

Lines show pairwise Sorensen dissimilarities of Darwin’s finch gut microbiome samples determined by time slices every 10 Mya correlated to pairwise dietary distances calculated with first foraging observations (red), pairwise dietary distances using 𝛿^13^C and 𝛿^15^N stable isotope measurements (green), and pairwise host phylogenetic distances (blue).


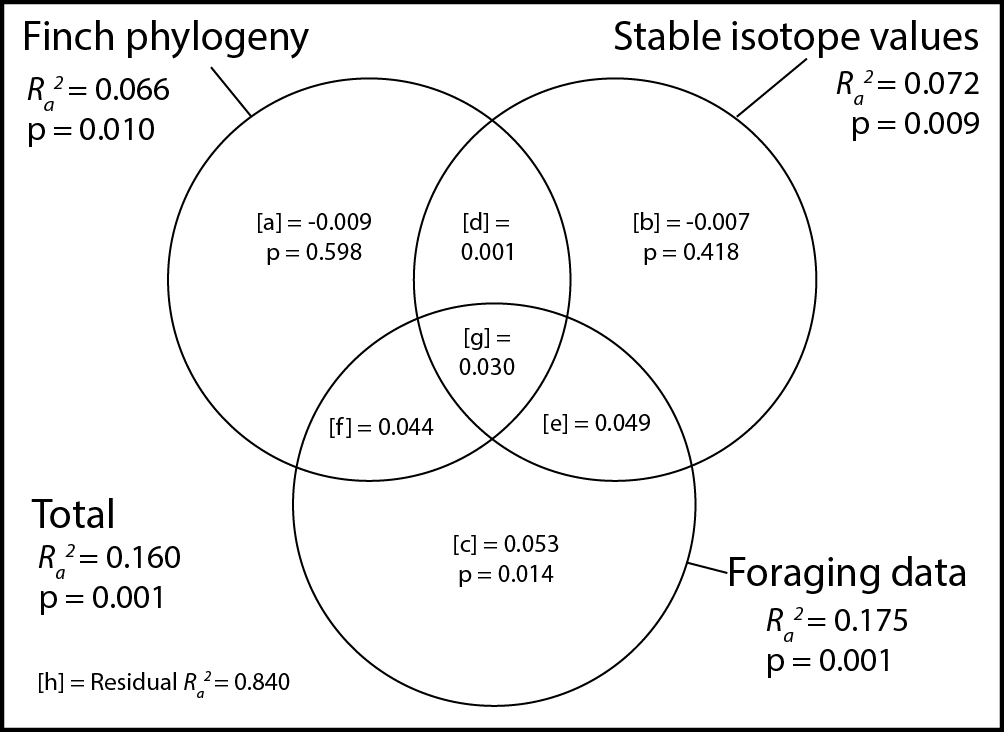


Figure S7. Variation partitioning of Darwin’s finch gut microbiome samples by finch phylogeny, stable isotope values, and foraging data.

Results of variation partitioning using weighted UniFrac distances between gut microbiome samples against Darwin’s finch phylogeny (first two principal coordinate axes), stable isotope values (𝛿^13^C and 𝛿^15^N values), and foraging data (first two principal component axes) visualized with a Venn diagram. Adjusted R^2^ values for each component are plotted inside the circles. All testable components include the p-value calculated using distance based redundancy analysis. Adjusted R^2^ values in [a], [b], and [c] are the amount of variation explained uniquely by the corresponding explanatory table. Parts [d], [e], and [f] are amounts of variation that can be explained by either table in the overlap and part [g] is shared by all three tables. Foraging data is the only table with a positive adjusted R^2^ value after controlling for overlapping variance.

| A | | Predicted Class | | | | | | | | | | | | | | | | | | | |  |
| --- | --- | --- | --- | --- | --- | --- | --- | --- | --- | --- | --- | --- | --- | --- | --- | --- | --- | --- | --- | --- | --- | --- |
|  |  | CF | | LGF | | LTF | | MGF | | SGF | | STF | | VF | | WF | | WPF | | Accuracy | | |
| Actual class | CF | | 0 | | 1 | | 0 | | 2 | | 3 | | 0 | | 0 | | 0 | | 0 | | 0.00 | |
|  | LGF | | 2 | | 0 | | 0 | | 4 | | 1 | | 0 | | 0 | | 0 | | 0 | | 0.00 | |
|  | LTF | | 1 | | 0 | | 0 | | 0 | | 0 | | 1 | | 0 | | 1 | | 0 | | 0.00 | |
|  | MGF | | 0 | | 3 | | 0 | | 0 | | 2 | | 0 | | 1 | | 0 | | 0 | | 0.00 | |
|  | SGF | | 2 | | 2 | | 0 | | 0 | | 8 | | 1 | | 0 | | 0 | | 0 | | 0.62 | |
|  | STF | | 0 | | 0 | | 0 | | 0 | | 2 | | 3 | | 0 | | 1 | | 1 | | 0.43 | |
|  | VF | | 2 | | 0 | | 0 | | 0 | | 2 | | 0 | | 0 | | 0 | | 0 | | 0.00 | |
|  | WF | | 0 | | 0 | | 0 | | 0 | | 3 | | 1 | | 0 | | 1 | | 1 | | 0.17 | |
|  | WPF | | 0 | | 0 | | 0 | | 0 | | 0 | | 1 | | 0 | | 3 | | 1 | | 0.20 | |

| B |  | Predicted class | | |
| --- | --- | --- | --- | --- |
|  |  | Highland | Lowland | Accuracy |
| Actual class | Highland | 27 | 2 | 0.93 |
|  | Lowland | 0 | 28 | 1.00 |

Figure S8. Confusion matrices for the random forest classifier using Darwin’s finch gut microbiome communities with leave-one-out cross-validation.

A) Prediction of finch species. Overall accuracy of the predictions was 0.23 and was equal to the no information rate. B) Prediction of habitat. Overall accuracy was 0.96 with a 95% confidence interval of 0.88 to 1.00. This was significantly higher than the no information rate (0.51, p<0.0001).

Figure S9. Abundance of the actual sequence variant with the most importance for classification by habitat, Tetrasphaera.

This ASV is present in almost all of the lowland samples but absent from almost all highland samples. This strong pattern of presence/absence explains why this ASV was so useful in classifying gut microbiome samples as coming from the highland or lowland habitats.

Supplementary Tables

Table S1. Statistical tests on amplicon library size mean and distribution across categorical variables of interest

| **Variable** | **Kruskal-Wallis test**  **for library size mean** | | **Levene’s test for**  **library size distribution** | |
| --- | --- | --- | --- | --- |
|  | **𝜲^2^** | **p-value** | **F value** | **p-value** |
| Species | 13.17 | 0.11 | 0.58 | 0.79 |
| Habitat | 0.11 | 0.74 | 0.05 | 0.82 |
| Sex | 0.51 | 0.48 | 2.23 | 0.14 |
| PCRPlate | 6.75 | 0.24 | 1.52 | 0.20 |

Table S2. Relative abundance of bacterial phyla across all samples

| **Phylum** | **meanRA** | **sdRA** | **minRA** | **maxRA** |
| --- | --- | --- | --- | --- |
| Firmicutes | 35.4% | 32.3% | 0.2% | 98.3% |
| Actinobacteria | 31.3% | 24.2% | 0.2% | 89.9% |
| Proteobacteria | 27.5% | 22.7% | 0.7% | 93.9% |
| Unclassified | 3.6% | 7.2% | 0.1% | 38.2% |
| Chloroflexi | 0.9% | 1.6% | 0.0% | 8.9% |
| Tenericutes | 0.4% | 2.3% | 0.0% | 17.2% |
| Acidobacteria | 0.3% | 0.4% | 0.0% | 1.6% |
| Planctomycetes | 0.3% | 0.3% | 0.0% | 1.5% |
| Bacteroidetes | 0.2% | 0.9% | 0.0% | 7.0% |
| Cyanobacteria | 0.1% | 0.2% | 0.0% | 1.1% |
| Spirochaetes | 0.1% | 0.4% | 0.0% | 2.8% |
| Deinococcus-Thermus | 0.0% | 0.1% | 0.0% | 0.7% |
| Verrucomicrobia | 0.0% | 0.0% | 0.0% | 0.2% |
| Chlamydiae | 0.0% | 0.0% | 0.0% | 0.0% |
| Gemmatimonadetes | 0.0% | 0.0% | 0.0% | 0.1% |

| **Phylum** | **CF** | **LGF** | **SGF** | **MGF** | **STF** | **LTF** | **WPF** | **VF** | **WF** |
| --- | --- | --- | --- | --- | --- | --- | --- | --- | --- |
| Firmicutes | 81.0% | 12.6% | 53.1% | 34.6% | 34.2% | 35.2% | 15.9% | 12.4% | 18.9% |
| Actinobacteria | 9.8% | 53.2% | 24.5% | 40.6% | 38.6% | 35.3% | 28.5% | 13.9% | 27.9% |
| Proteobacteria | 7.4% | 27.2% | 19.6% | 15.9% | 24.4% | 28.8% | 48.1% | 54.9% | 43.7% |
| Unclassified | 1.1% | 4.0% | 1.5% | 6.1% | 0.7% | 0.3% | 1.3% | 17.3% | 6.0% |
| Chloroflexi | 0.3% | 2.2% | 0.5% | 2.0% | 1.3% | 0.3% | 0.4% | 0.1% | 1.1% |
| Tenericutes | 0.1% | 0.0% | 0.0% | 0.0% | 0.0% | 0.0% | 3.7% | 0.0% | 0.9% |
| Acidobacteria | 0.1% | 0.3% | 0.3% | 0.5% | 0.4% | 0.0% | 0.2% | 0.3% | 0.2% |
| Planctomycetes | 0.1% | 0.2% | 0.3% | 0.3% | 0.4% | 0.1% | 0.3% | 0.1% | 0.4% |
| Bacteroidetes | 0.1% | 0.0% | 0.0% | 0.0% | 0.1% | 0.0% | 1.5% | 0.2% | 0.0% |
| Cyanobacteria | 0.0% | 0.1% | 0.1% | 0.0% | 0.1% | 0.0% | 0.1% | 0.5% | 0.1% |
| Spirochaetes | 0.0% | 0.0% | 0.0% | 0.0% | 0.0% | 0.0% | 0.0% | 0.0% | 0.7% |
| Deinococcus-Thermus | 0.0% | 0.0% | 0.0% | 0.0% | 0.0% | 0.0% | 0.0% | 0.3% | 0.0% |

Table S3. Relative abundance of bacterial phyla across Darwin’s finch species

Table S4. Most abundant bacterial genera across all Darwin finch gut microbiome samples above 1 percent mean relative abundance.

| **Phylum** | **Genus** | **meanRA** | **sdRA** | **minRA** | **maxRA** |
| --- | --- | --- | --- | --- | --- |
| Firmicutes | Lactobacillus | 26.2% | 32.8% | 0.0% | 98.2% |
| Proteobacteria | Acinetobacter | 6.4% | 16.0% | 0.0% | 86.0% |
| Actinobacteria | Kocuria | 4.5% | 12.2% | 0.0% | 69.7% |
| Proteobacteria | Methylobacterium | 3.8% | 4.9% | 0.0% | 25.6% |
| Actinobacteria | Mycobacterium | 2.6% | 6.6% | 0.0% | 51.3% |
| Actinobacteria | Nocardioides | 1.8% | 1.9% | 0.0% | 9.0% |
| Actinobacteria | Geodermatophilus | 1.8% | 3.2% | 0.0% | 16.3% |
| Proteobacteria | Diplorickettsia | 1.7% | 11.3% | 0.0% | 89.5% |
| Actinobacteria | Solirubrobacter | 1.7% | 2.1% | 0.0% | 9.1% |
| Firmicutes | Enterococcus | 1.7% | 4.6% | 0.0% | 22.6% |
| Actinobacteria | Cellulomonas | 1.4% | 3.1% | 0.0% | 21.3% |
| Proteobacteria | Enterobacter | 1.3% | 4.8% | 0.0% | 29.8% |
| Actinobacteria | Curtobacterium | 1.3% | 2.6% | 0.0% | 13.5% |
| Proteobacteria | Rhodospirillum | 1.3% | 9.9% | 0.0% | 78.9% |
| Proteobacteria | Rhizobium | 1.2% | 1.6% | 0.0% | 7.7% |
| Firmicutes | Clostridium_XVIII | 1.1% | 8.4% | 0.0% | 67.0% |
| Actinobacteria | Corynebacterium | 1.0% | 5.0% | 0.0% | 39.6% |

Table S5. Most abundant classified bacterial genus for each Darwin finch species

| **Darwin finch species** | **Bacterial genus** | **meanRA** | **sdRA** | **minRA** | **maxRA** |
| --- | --- | --- | --- | --- | --- |
| SGF | Lactobacillus | 49.7% | 34.3% | 0.2% | 95.7% |
| MGF | Lactobacillus | 27.3% | 34.8% | 0.1% | 84.5% |
| LGF | Kocuria | 22.5% | 26.5% | 2.0% | 69.7% |
| CF | Lactobacillus | 77.8% | 16.3% | 56.5% | 98.2% |
| STF | Lactobacillus | 11.7% | 20.3% | 0.1% | 61.8% |
| LTF | Lactobacillus | 26.1% | 13.2% | 14.0% | 40.2% |
| WPF | Rhodospirillum | 15.8% | 35.3% | 0.0% | 78.9% |
| VF | Acinetobacter | 16.5% | 29.9% | 1.3% | 61.4% |
| WF | Diplorickettsia | 14.0% | 33.4% | 0.1% | 89.5% |

Table S6. Alpha diversity estimates by Darwin’s finch species

| **Species** | **Observed ASVs mean** | **Observed ASVs SE** | **Chao1 mean** | **Chao1 SE** | **PD mean** | **PD SE** |
| --- | --- | --- | --- | --- | --- | --- |
| CF | 522.17 | 151.25 | 701.04 | 188.83 | 52.09 | 10.04 |
| LGF | 796.63 | 112.25 | 1023.84 | 110.62 | 66.04 | 6.54 |
| SGF | 622.46 | 113.56 | 853.22 | 131.87 | 53.60 | 6.08 |
| WF | 575.00 | 70.67 | 729.71 | 74.16 | 59.64 | 4.99 |
| STF | 777.50 | 88.13 | 962.39 | 101.93 | 68.51 | 6.56 |
| MGF | 732.43 | 141.23 | 928.88 | 151.93 | 59.02 | 7.29 |
| VF | 471.25 | 73.26 | 623.35 | 84.95 | 58.62 | 4.80 |
| LTF | 475.67 | 151.15 | 684.07 | 170.49 | 44.06 | 8.20 |
| WPF | 997.20 | 99.30 | 1228.33 | 127.62 | 80.87 | 5.58 |

Table S7. Anova on alpha diversity metrics across species (N = 9, sample size ranging from 3 to 13)

| Diversity metric | F value | p value |
| --- | --- | --- |
| Observed ASVs | 1.58 | 0.15 |
| Chao 1 | 1.50 | 0.18 |
| Phylogenetic diversity | 1.67 | 0.13 |

Table S8. Permanova tests of weighted UniFrac distances with categorical variables of interest (N = 62)

| **Variable** | **F** | **R^2^** | **p** |
| --- | --- | --- | --- |
| Habitat* | 12.1 | 0.15 | 0.03 |
| Habitat:Species* | 1.6 | 0.21 | 0.03 |
| Sex | 0.97 | 0.02 | 0.41 |
| PCRPlate | 0.78 | 0.01 | 0.56 |

* Species is nested within habitat because only the small ground finch is present in both habitats

Table S9. *Post hoc* pairwise anova with weighted UniFrac distances between Darwin’s finch species

| **Pairwise comparison** | **F.Model** | **R2** | **p.value** | **p.adjusted** |
| --- | --- | --- | --- | --- |
| WF vs VF | 6.46 | 0.45 | 0.005 | 0.180 |
| WF vs STF | 1.95 | 0.12 | 0.020 | 0.720 |
| WF vs SGF | 1.75 | 0.09 | 0.130 | 1.000 |
| WF vs LGF | 4.66 | 0.28 | 0.001 | *0.036 |
| WF vs LTF | 2.49 | 0.26 | 0.052 | 1.000 |
| WF vs MGF | 2.79 | 0.20 | 0.013 | 0.468 |
| WF vs WPF | 1.75 | 0.16 | 0.025 | 0.900 |
| WF vs CF | 3.49 | 0.26 | 0.005 | 0.180 |
| VF vs STF | 5.28 | 0.31 | 0.001 | *0.036 |
| VF vs SGF | 2.43 | 0.14 | 0.047 | 1.000 |
| VF vs LGF | 4.28 | 0.30 | 0.006 | 0.216 |
| VF vs LTF | 1.88 | 0.27 | 0.046 | 1.000 |
| VF vs MGF | 3.07 | 0.25 | 0.044 | 1.000 |
| VF vs WPF | 6.37 | 0.48 | 0.007 | 0.252 |
| VF vs CF | 1.12 | 0.12 | 0.306 | 1.000 |
| STF vs SGF | 1.78 | 0.08 | 0.096 | 1.000 |
| STF vs LGF | 4.64 | 0.22 | 0.001 | *0.036 |
| STF vs LTF | 2.23 | 0.17 | 0.048 | 1.000 |
| STF vs MGF | 2.71 | 0.15 | 0.017 | 0.612 |
| STF vs WPF | 1.65 | 0.11 | 0.083 | 1.000 |
| STF vs CF | 3.76 | 0.21 | 0.002 | 0.072 |
| SGF vs LGF | 2.04 | 0.10 | 0.075 | 1.000 |
| SGF vs LTF | 0.90 | 0.06 | 0.398 | 1.000 |
| SGF vs MGF | 1.22 | 0.06 | 0.298 | 1.000 |
| SGF vs WPF | 1.85 | 0.10 | 0.088 | 1.000 |
| SGF vs CF | 1.40 | 0.08 | 0.212 | 1.000 |
| LGF vs LTF | 3.37 | 0.27 | 0.023 | 0.828 |
| LGF vs MGF | 0.67 | 0.05 | 0.727 | 1.000 |
| LGF vs WPF | 3.73 | 0.25 | 0.004 | 0.144 |
| LGF vs CF | 1.88 | 0.14 | 0.107 | 1.000 |
| LTF vs MGF | 1.72 | 0.18 | 0.155 | 1.000 |
| LTF vs WPF | 2.97 | 0.33 | 0.049 | 1.000 |
| LTF vs CF | 1.13 | 0.14 | 0.271 | 1.000 |
| MGF vs WPF | 2.27 | 0.19 | 0.039 | 1.000 |
| MGF vs CF | 1.22 | 0.10 | 0.261 | 1.000 |
| WPF vs CF | 3.20 | 0.26 | 0.008 | 0.288 |

Table S10. Stable isotope (𝛿 ^13^C and 𝛿 ^15^N) ratios by Darwin’s finch species

| **Species** | **Habitat** | **𝛿 ^13^C mean (‰)** | **𝛿 ^13^C SD (‰)** | **𝛿 ^15^N mean (‰)** | **𝛿 ^15^N SD (‰)** |
| --- | --- | --- | --- | --- | --- |
| SGF | H | -20.9 | 5.3 | 6.9 | 1.5 |
|  | L | -23.6 | 0.7 | 10.1 | 1.8 |
| MGF | H | -24.6 | -* | 8.7 | -* |
|  | L | -23.0 | 2.0 | 10.1 | 1.1 |
| LGF | L | -23.3 | 1.0 | 9.3 | 0.7 |
| CF | L | -16.2 | 1.6 | 11.4 | 1.2 |
| STF | H | -25.2 | 1.4 | 7.7 | 1.0 |
|  | L | -23.7 | -* | 12.9 | -* |
| LTF | H | -25.7 | 0.2 | 8.9 | 0.5 |
| WPF | H | -25.6 | 0.8 | 9.2 | 0.8 |
| VF | L | -24.5 | 0.2 | 10.5 | 1.0 |
| WF | H | -26.7 | 0.7 | 9.3 | 0.3 |

* Single sample for this species/habitat so standard deviation was not calculated

Table S11 Kruskal-Wallis tests of stable isotope ratios

| Stable isotope | Variable | Kruskal-Wallis chi-squared | p |
| --- | --- | --- | --- |
| **𝛿 ^13^C** | Habitat | 16.3 | 5.5e-05 |
|  | Species | 34.8 | 2.9e-05 |
| **𝛿 ^15^N** | Habitat | 23.0 | 1.6e-05 |
|  | Species | 22.5 | 0.004 |

**Table S12 Dunn Test on 𝛿 ^13^C by species.**

| Comparison | Z | P.unadj | P.adj* |
| --- | --- | --- | --- |
| CF - LGF | 1.747 | 0.081 | 1.000 |
| CF - LTF | 3.211 | 0.001 | **0.048** |
| LGF - LTF | 1.882 | 0.060 | 1.000 |
| CF - MGF | 2.016 | 0.044 | 1.000 |
| LGF - MGF | 0.280 | 0.779 | 1.000 |
| LTF - MGF | -1.665 | 0.096 | 1.000 |
| CF - SGF | 2.185 | 0.029 | 1.000 |
| LGF - SGF | 0.228 | 0.820 | 1.000 |
| LTF - SGF | -1.861 | 0.063 | 1.000 |
| MGF - SGF | -0.091 | 0.927 | 1.000 |
| CF - STF | 3.506 | 0.000 | **0.016** |
| LGF - STF | 1.781 | 0.075 | 1.000 |
| LTF - STF | -0.557 | 0.577 | 1.000 |
| MGF - STF | 1.492 | 0.136 | 1.000 |
| SGF - STF | 1.813 | 0.070 | 1.000 |
| CF - VF | 2.533 | 0.011 | 0.407 |
| LGF - VF | 1.058 | 0.290 | 1.000 |
| LTF - VF | -0.832 | 0.405 | 1.000 |
| MGF - VF | 0.819 | 0.413 | 1.000 |
| SGF - VF | 0.973 | 0.330 | 1.000 |
| STF - VF | -0.422 | 0.673 | 1.000 |
| CF - WF | 4.857 | 0.000 | **4.2e-05b** |
| LGF - WF | 3.294 | 0.001 | **0.036** |
| LTF - WF | 0.755 | 0.450 | 1.000 |
| MGF - WF | 3.025 | 0.002 | 0.090 |
| SGF - WF | 3.497 | 0.000 | 0.017 |
| STF - WF | 1.687 | 0.092 | 1.000 |
| VF - WF | 1.811 | 0.070 | 1.000 |
| CF - WPF | 3.734 | 0.000 | **0.007** |
| LGF - WPF | 2.202 | 0.028 | 0.997 |
| LTF - WPF | -0.013 | 0.989 | 1.000 |
| MGF - WPF | 1.946 | 0.052 | 1.000 |
| SGF - WPF | 2.247 | 0.025 | 0.887 |
| STF - WPF | 0.645 | 0.519 | 1.000 |
| VF - WPF | 0.933 | 0.351 | 1.000 |
| WF - WPF | -0.897 | 0.369 | 1.000 |

*****p-values adjusted by Bonferroni correction

**Table S13 Dunn Test on 𝛿 ^15^N by species.**

| Comparison | Z | P.unadj | P.adj* |
| --- | --- | --- | --- |
| CF - LGF | 2.088 | 0.037 | 1.000 |
| CF - LTF | 2.209 | 0.027 | 0.977 |
| LGF - LTF | 0.581 | 0.562 | 1.000 |
| CF - MGF | 1.483 | 0.138 | 1.000 |
| LGF - MGF | -0.630 | 0.529 | 1.000 |
| LTF - MGF | -1.069 | 0.285 | 1.000 |
| CF - SGF | 3.708 | 0.000 | **0.008** |
| LGF - SGF | 1.426 | 0.154 | 1.000 |
| LTF - SGF | 0.418 | 0.676 | 1.000 |
| MGF - SGF | 2.145 | 0.032 | 1.000 |
| CF - STF | 3.616 | 0.000 | **0.011** |
| LGF - STF | 1.529 | 0.126 | 1.000 |
| LTF - STF | 0.577 | 0.564 | 1.000 |
| MGF - STF | 2.180 | 0.029 | 1.000 |
| SGF - STF | 0.273 | 0.785 | 1.000 |
| CF - VF | 0.556 | 0.578 | 1.000 |
| LGF - VF | -1.281 | 0.200 | 1.000 |
| LTF - VF | -1.575 | 0.115 | 1.000 |
| MGF - VF | -0.743 | 0.457 | 1.000 |
| SGF - VF | -2.573 | 0.010 | 0.363 |
| STF - VF | -2.603 | 0.009 | 0.333 |
| CF - WF | 1.983 | 0.047 | 1.000 |
| LGF - WF | -0.030 | 0.976 | 1.000 |
| LTF - WF | -0.590 | 0.555 | 1.000 |
| MGF - WF | 0.576 | 0.565 | 1.000 |
| SGF - WF | -1.388 | 0.165 | 1.000 |
| STF - WF | -1.496 | 0.135 | 1.000 |
| VF - WF | 1.218 | 0.223 | 1.000 |
| CF - WPF | 2.112 | 0.035 | 1.000 |
| LGF - WPF | 0.200 | 0.841 | 1.000 |
| LTF - WPF | -0.388 | 0.698 | 1.000 |
| MGF - WPF | 0.776 | 0.438 | 1.000 |
| SGF - WPF | -1.048 | 0.295 | 1.000 |
| STF - WPF | -1.182 | 0.237 | 1.000 |
| VF - WPF | 1.371 | 0.170 | 1.000 |
| WF - WPF | 0.221 | 0.825 | 1.000 |

*****p-values adjusted by Bonferroni correction

Table S14. First foraging observations across all Darwin’s finch species and both habitats

| Sp. | Hab | Total (n) | Counts (n) | | | | | Proportion of counts (%) | | | | | Summary (%) | |
| --- | --- | --- | --- | --- | --- | --- | --- | --- | --- | --- | --- | --- | --- | --- |
|  |  |  | Flower | Fruit | Leaf | Seed | Insect | Flower | Fruit | Leaf | Seed | Insect | Plant* | Insect |
| SGF | L | 12 | 2 | - | - | 6 | 4 | 17 | - | - | 50 | 33 | 67 | 33 |
|  | H | 25 | - | 3 | - | 15 | 7 | - | 12 | - | 60 | 28 | 72 | 28 |
| MGF | L | 20 | 5 | - | 2 | 12 | 1 | 25 | - | 10 | 60 | 5 | 95 | 5 |
|  | H | 12 | - | - | - | 6 | 6 | - | - | - | 50 | 50 | 50 | 50 |
| LGF | L | 11 | - | - | 1 | 9 | 1 | - | - | 9 | 82 | 9 | 91 | 9 |
| CF | L | 6 | 3 | - | 3 | - | - | 50 | - | 50 | - | - | 100 | 0 |
| STF | H | 41 | - | - | - | 4 | 37 | - | - | - | 10 | 90 | 10 | 90 |
| LTF | H | 15 | - | 3 | - | - | 12 | - | 20 | - | - | 80 | 20 | 80 |
| WPF | H | 19 | - | - | - | - | 19 | - | - | - | - | 100 | 0 | 100 |
| VF | L | 6 | 2 | - | 4 | - | - | 33 | - | 67 | - | - | 100 | 0 |
| WF | H | 34 | - | - | - | - | 34 | - | - | - | - | 100 | 0 | 100 |

* The category ‘Plant’ is the sum of all plant derived food items (flower, fruit, leaf, and seed).

Table S15. Procrustes Analysis of Co-Phylogeny.

|  | **Host phylogeny** | **Stable isotope*** | **Diet**** |
| --- | --- | --- | --- |
| R^2^ | 0.178 | 0.064 | 0.175 |
| p-value | <0.0001 | 0.013 | <0.0001 |

* Euclidean distances were calculated between δ^13^C and δ^15^N values for all microbiome samples

** Euclidean distances were calculated based on proportion of food items in first foraging observations

Table S16. Top actual sequence variants (ASVs) for classifying gut microbiome samples by habitat of origin using the random forest algorithm

| Phylum | Family | Genus | meanMDA* |
| --- | --- | --- | --- |
| Actinobacteria | Intrasporangiaceae | Tetrasphaera | 0.009 |
| Actinobacteria | Mycobacteriaceae | Mycobacterium | 0.008 |
| Actinobacteria | Nocardiaceae | Williamsia | 0.008 |
| Actinobacteria | Micromonosporaceae | Unclassified | 0.008 |
| Actinobacteria | Patulibacteraceae | Patulibacter | 0.007 |
| Proteobacteria | Methylobacteriaceae | Methylobacterium | 0.006 |
| Actinobacteria | Mycobacteriaceae | Mycobacterium | 0.005 |
| Actinobacteria | Mycobacteriaceae | Mycobacterium | 0.005 |
| Actinobacteria | Mycobacteriaceae | Mycobacterium | 0.005 |
| Actinobacteria | Nocardioidaceae | Nocardioides | 0.005 |

* Mean decrease in accuracy averaged across all 62 random forest classifiers
